# COVID-19 in Vietnam and Its Impact on Road Trauma: Retrospective Study Based on National Data

**DOI:** 10.2196/40883

**Published:** 2023-02-06

**Authors:** Ba Tuan Nguyen, Christopher Leigh Blizzard, Andrew Palmer, Huu Tu Nguyen, Thang Cong Quyet, Viet Tran, Mark Nelson

**Affiliations:** 1 Menzies Institute for Medical Research University of Tasmania Hobart Australia; 2 Hanoi Medical University Hanoi Vietnam; 3 Tasmanian School of Medicine University of Tasmania Hobart Australia; 4 Tasmanian Health Service Hobart Australia

**Keywords:** COVID-19, impact, road trauma, low- and middle-income country, LMIC, mortality, pandemic, trauma, social distancing, lockdown, Vietnam, disease, policy, deaths

## Abstract

**Background:**

Despite significant improvement in the last decade, road trauma remains a substantial contributor to deaths in Vietnam. The COVID-19 pandemic necessitated public health measures that had an unforeseen benefit on road trauma in high-income countries. We investigate if this reduction was also seen in a low- to middle-income country like Vietnam.

**Objective:**

Our aim was to investigate how the COVID-19 pandemic and the government policies implemented in response to it impacted road trauma fatalities in Vietnam. We also compared this impact to other government policies related to road trauma implemented in the preceding 14 years (2007-2020).

**Methods:**

COVID-19 data were extracted from the Vietnamese Ministry of Health database. Road traffic deaths from 2007 to 2021 were derived from the Vietnamese General Statistical Office. We used Stata software (version 17; StataCorp) for statistical analysis. Poisson regression modeling was used to estimate trends in road fatality rates based on annual national mortality data for the 2007-2021 period. The actual change in road traffic mortality in 2021 was compared with calculated figures to demonstrate the effect of COVID-19 on road trauma fatalities. We also compared this impact to other government policies that aimed to reduce traffic-related fatalities from 2007 to 2020.

**Results:**

Between 2007 and 2020, the number of annual road traffic deaths decreased by more than 50%, from 15.3 to 7 per 100,000 population, resulting in an average reduction of 5.4% per annum. We estimated that the road traffic mortality rate declined by 12.1% (95% CI 8.9-15.3%) in 2021 relative to this trend. The actual number of road trauma deaths fell by 16.4%. This reduction was largely seen from August to October 2021 when lockdown and social distancing measures were in force.

**Conclusions:**

In 2021, the road traffic–related death reduction in Vietnam was 3 times greater than the trend seen in the preceding 14 years. The public health response to the COVID-19 pandemic in Vietnam was associated with a third of this reduction. It can thus be concluded that government policies implemented to address the COVID-19 pandemic resulted in a 4.3% decrease in road traffic deaths in 2021. This has been observed in high-income countries, but we have demonstrated this for the first time in a low- and middle-income country.

## Introduction

### Background

COVID-19 is an infectious disease caused by the SARS-CoV-2 virus, which was discovered in Wuhan, China, on December 31, 2019 [1]. In Vietnam, the first confirmed COVID-19 case was recorded in late January 2020 [2]. During that year, while most countries around the world struggled against the pandemic, Vietnam was considered one of the safest places in terms of COVID-19 community transmission [3]. This was due to a strict “zero-COVID” government policy that included closure of the Vietnam-China border, restrictions to air travel from any affected countries, quarantining of infected patients and all close contacts, mandatory use of masks in public places, closure of all nonessential services, and use of rapid antigen or real-time–polymerase chain reaction testing for all suspected cases [4]. However, this changed quickly in the following year. While most affected countries were reopening, a wave of COVID-19 spread across Vietnam. There were 4 waves of COVID-19 outbreak in Vietnam [5], but the fourth outbreak, which started in April 2021 and was caused by the Delta variant, was the most catastrophic [6]. To control this situation, as was done in other countries [7], social distancing and lockdown strategies were applied in Vietnam by the central government to local government areas according to their disease burden [8]. The Vietnamese government placed approximately a third of the national population (including the 2 largest cities, Hanoi and Ho Chi Minh City) under lockdown starting on July 23, 2021. This lasted for about 3 months and was gradually relaxed in November 2021 as the pandemic was brought under control.

Another contemporary “pandemic” is road trauma, which has been a significant contributor to morbidity and mortality in Vietnam and other low- and middle-income countries (LMICs) for decades [9]. The risk of road traffic death in LMICs over the last decade was more than 3 times higher than in high-income countries (HICs) such as the United States, United Kingdom, and Australia [10]. In the last 15 years in Vietnam, approximately 9000 people per annum have been killed in road accidents, with an equal number hospitalized [11,12]. This problem has been driven by poor infrastructure and the dominance of the motorcycle as the mode of transport, often overladen with passengers and cargo [9]. To address this problem, the Vietnamese government legislated multiple public health measures. In 2008, helmets became mandatory for all motorcycle riders and their passengers [13]. Mandatory seatbelt use in other vehicles was introduced in the same year [13]. Media campaigns and public traffic law education have also been ubiquitous since this time. Subsequently, road deaths have steadily declined [14].

The lockdown and social distancing measures were implemented to reduce transmission and death due to COVID-19 but may have had an unforeseen benefit on road trauma. For example, social distancing may limit the number of passengers in an individual vehicle and, hence, reduce the number of people exposed to trauma should that vehicle crash. Lockdowns also reduce exposure since population movements are limited to essential travel and cross-border movements are restricted. Reductions have been seen in most HICs [15]. For example, in Australia where such interventions were widely enforced, there was a 7% reduction in road trauma deaths in 2020 compared with 2019 [15-17]. Our study aimed to investigate if those reductions were also seen in an LMIC like Vietnam.

### Objective

We aimed to investigate the effect of the COVID-19 pandemic and the Vietnamese government’s policies in response to it on road trauma fatalities. We also compared this impact with other improvements related to government policies over the past 14 years (2007-2020) regarding road trauma management.

## Methods

### Data Sources

COVID-19 data were extracted from the Vietnamese Ministry of Health database [18]. Road traffic mortality (2007-2021) was derived from the Vietnamese General Statistical Office [19]. All data sources were anonymous or deidentified for privacy and confidentiality protection.

### Statistical Methodology

We used Stata software (version 17; StataCorp) for statistical analysis. Poisson regression modeling, with the logarithm of the national population of Vietnam for each year included as an offset, was used to estimate trends in the rates of road fatalities from the annual national mortality data for the 2007-2021 period. The Poisson regression model related the expected number *E*(*Y_t_*) of road fatalities *Y_t_* in Vietnam in each year *t* (*t*=2007, 2008,…, 2021) to a vector of covariates **x***_t_* for that year according to the log-linear form:




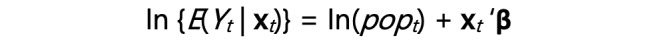




where *pop_t_* is the estimated national population of Vietnam for year *t* and In(*pop_t_*) is its logarithm entered as an offset (a covariate with a coefficient of unity), and 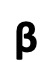 is the vector of coefficients to be estimated. The final form of the linear predictor **x***_t_* 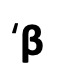 is as follows:




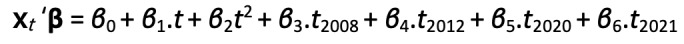




where t_2008_, t_2012_, t_2020_, and t_2021_ are step functions that allow the vertical position of the quadratic line of best fit to shift up or down (a level change) in the years nominated (2008, 2012, 2020, and 2021) and subsequent years. For example, the step function t_2008_ was defined as:




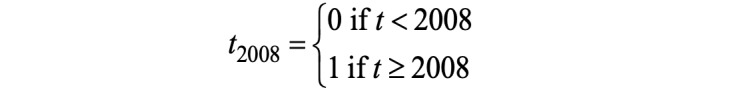




The exponential value of the estimated coefficient 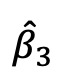 is an estimate of the percentage change (step up or step down) that occurred in the year 2008. The other step functions were defined in analogous ways. The 4 years involved were chosen a priori as the years that helmet and seatbelt use were made mandatory (2008), traffic safety regulations were strengthened (2012), impaired driving laws were fortified (2020), and the COVID-19 response was implemented (2021). Having annual data only, there was neither capacity nor need to estimate the lag in months between implementation and impact, nor was it necessary to use segmented regression methods. The final form of the linear predictor was selected after fitting interaction terms between each of the step functions and the covariates (*t* and *t*^2^) for year to allow the slope of the trend line to change. In every case, the null hypothesis that no change in slope had occurred was accepted (*P*>.05). There was no evidence of extra-Poisson variation (deviance goodness-of-fit test: *P*=.13) or of autocorrelation in the residuals (Q-statistic: *P*>.05 at each lag). The actual change in road traffic mortality in 2021 was compared with calculated figures to demonstrate the effect of COVID-19 measures on road trauma fatality.

### Ethical Considerations

The study was approved for human ethics exemption (H0027318 [H-84839]), issued on April 6, 2022, by the Human Research Ethics Committee of the University of Tasmania.

## Results

### The COVID-19 Pandemic in Vietnam

**Figure 1:**
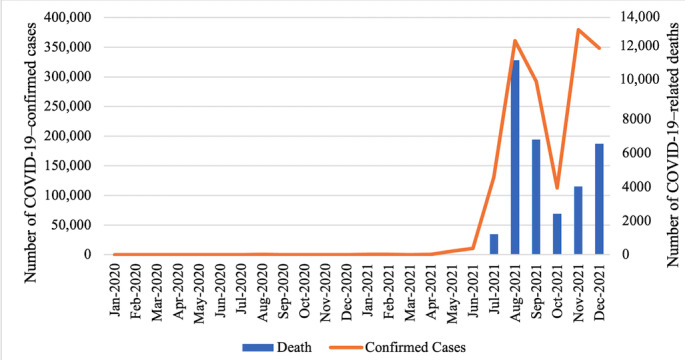
Deaths and confirmed cases of COVID-19 in Vietnam from 2020 to 2021 [18].

There were fewer than 3000 confirmed COVID-19 cases and 35 COVID-19–related deaths during the initial 15 months since the first recorded case in January 2020. However, with the appearance of the Delta variant, the number of confirmed cases escalated exponentially from 373 in April 2021 to 10,730 in June 2021, reaching a peak of over 360,000 in September and a second peak in November (Figure 1). Similarly, the number of deaths due to COVID-19 rapidly escalated from 18 in May 2021 to 11,487 in August 2021. A high death rate persisted until the end of 2021 [6]. In response, the Vietnamese government imposed a lockdown (from July 23 to October 31) on approximately one-third of the country’s population (about 32 million people), including in its most populous cities, Hanoi and Ho Chi Minh City.

### Road Trauma Fatalities in Vietnam

**Figure 2:**
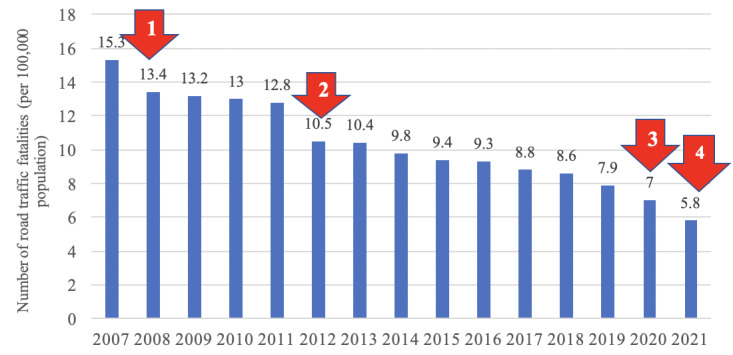
Number of road traffic fatalities per 100,000 population in Vietnam from 2007 to 2021 [12,13,20]. Arrows indicate government policies that were intentionally or otherwise implemented to address this issue: (1) mandatory helmet use, (2) strengthening of the national traffic safety committee (ie, increased presence of traffic police and increased financial penalties for traffic violations), (3) implementation of the zero-alcohol policy, and (4) COVID-19–related measures.

Between 2007 and 2020, the rate of road traffic fatalities decreased by more than 50% from 15.3 to 7 per 100,000 population, an average reduction of 5.4% per annum. The greatest improvements were seen in 2008, 2012, and 2020 (13.0%, 18.0%, and 11.4%, respectively; Figure 2). We estimated that road traffic mortality rates declined by 12.1% (95% CI 8.9-15.3%) in 2021 relative to this trend. In 2021, there were 5739 deaths, which was a 16.4% decrease from the previous year [20]. Thus, there was a further reduction of 4.3% in road trauma deaths in 2021 compared to the previous trend.

### COVID-19’s Impact on Road Trauma

**Figure 3:**
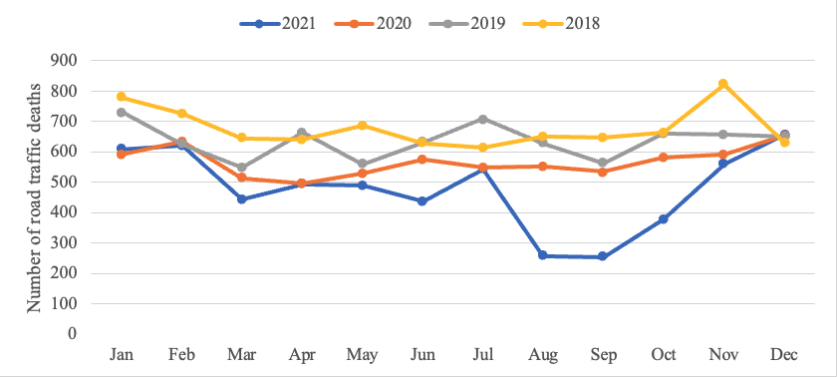
Monthly statistics on road accident fatalities from 2018 to 2021 in Vietnam [20].

During the first 2 calendar months of the lockdown, the number of traffic accident deaths decreased by more than 50% from 543 in July to 257 and 254 in August and September 2021, respectively (Figure 3). It then increased to 377 in October before increasing more substantially to 580 and 680 in November and December, respectively [21].

## Discussion

### Principal Findings

Since the declaration of COVID-19 as a global pandemic by the World Health Organization, the disease has swept through all countries. COVID-19 had minimal impact in Vietnam during the 15 months following its first confirmed case due to the early and decisive policies implemented by the Vietnamese government. However, these early successes were undone by the Delta variant after April 2021. The number of cases and deaths rapidly increased from then onward. Due to vaccine scarcity, the Vietnamese government had to initially rely on lockdown and social distancing policies to control the pandemic. With the availability of community vaccination and its high uptake, lockdown and social distancing measures were relaxed by November 2021. The number of COVID-19 cases reached a new peak, but the fatality rate was only a third of that in August 2021 [22]. According to data from the World Health Organization, countries worldwide have experienced these trends regardless of social economic standing [23].

### Road Trauma: A Steady Improvement in the Past 14 Years

Road trauma is a leading cause of death in Vietnam [13]. Like other LMICs, common causes include poor road safety and law enforcement, impaired driving, and speeding. Vietnam has been ranked 50 out of 183 recorded countries for road traffic deaths per 100,000 population [24]. Consequently, reducing road traffic deaths has been a priority of the Vietnamese government. A range of government decrees, regulations, laws, and policies have been issued and updated to improve the population’s health and financial circumstances. As a result of these initiatives, road trauma deaths have been decreasing by an average of 5.4% per annum over the 2007-2020 period, with the greatest reductions seen in 2008, 2012, and 2020 (Figure 1). The first reduction coincided with the implementation of mandatory motorcycle helmet use legislation [25]. The second was associated with the national traffic safety committee increasing traffic police presence on the road and enforcement of increased financial penalties for traffic violations [11]. The third was associated with the introduction of zero-alcohol laws for all drivers and passengers [26].

### A “Positive” Side of the Pandemic

We found that road trauma deaths in Vietnam in 2021 declined at a greater rate compared to the long-term trend. This was likely in response to the COVID-19 pandemic lockdown. In 2021, we found a 16.4 % improvement in road trauma deaths compared with an average 5.4% reduction over the preceding 14 years. This reduction fluctuated month to month. A reason for this distortion was that during the 3 months of lockdown, the number of vehicles on the road decreased by 84%, largely contributing to the total year reduction of 37% [27]. Likewise, Yasin et al [15] compared April 2020 to April 2019 and found that 32 out of 36 countries had a sharp drop in traffic volume, leading to a substantial reduction in road crashes and road trauma deaths.

It could be argued that the mortality rate was already declining due to other factors, and the additional decline beyond the preceding trend in 2021 (4.3%) was a delayed effect of the “zero-alcohol” policy from 2020 and not attributable to COVID-19. The temporal relationship of road trauma deaths in relation to the lockdown (Figure 3) supports our attribution beyond the zero-alcohol policy.

### Effects of the COVID-19 Pandemic on Road Trauma Deaths Globally

**Figure 4:**
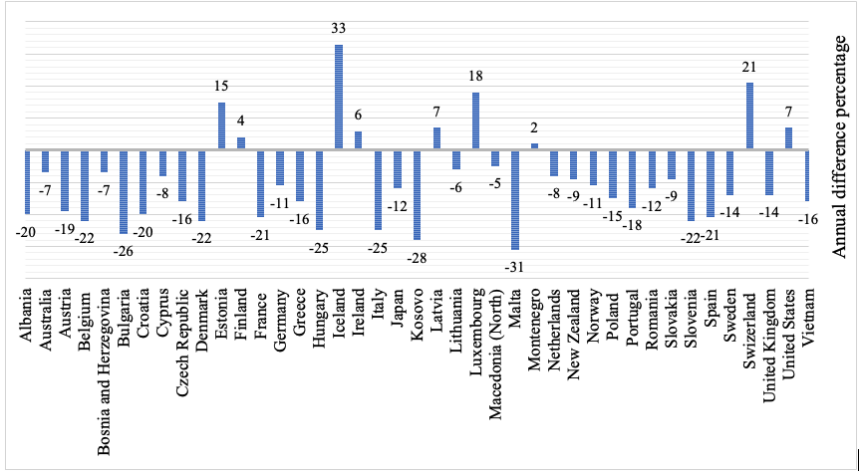
Comparison of the annual difference percentage in road trauma deaths prior to and during the COVID-19 pandemic in Vietnam and other countries [16,17,27-35].

A similar global trend was seen in most HICs where data have been published, with most countries showing a reduction in the annual road accident fatality rate (annual difference percentages) ranging from 5% in Macedonia to 31% in Malta. Some exceptions were seen in several countries with increases observed in Estonia (15%), Finland (4%), Iceland (33%), and the United States (7%) (Figure 4).

The explanation for this may be that these countries had not applied public health measures such as lockdowns with as much vigor or as extensively. There may also have been an increased use of private vehicles due to a fear of using public transport [36], leading to increased road accidents [26,27].

A lesson learned from the COVID-19 pandemic is that one approach to limiting road trauma deaths and injuries is to reduce vehicular traffic. Though this seems a trivial observation and interpretation, it does suggest that the Vietnamese government should prioritize the improvement of public transport such as bus and train systems to reduce private vehicle use and subsequently road trauma and deaths.

### Limitations

This study would have benefited from additional national and international comparisons. We were limited in performing this analysis due to dissimilarity between national and international data not only for LMICs but also for HICs [37]. In this paper, we did not use information from international sources such as the World Health Organization, the World Bank, or the United Nations since the data have been insufficient in the last 15 years and highly variable. For these reasons, we chose original data sourced from the Vietnamese General Statistical Office and Ministry of Health.

### Conclusion

In 2021, the reduction in road traffic deaths in Vietnam was 3 times greater than the trend over the preceding 14 years. The public health response to the COVID-19 pandemic in Vietnam was associated with this reduction. This has also been observed in HICs but was demonstrated by us for the first time in an LMIC.

## Abbreviations

HIC: high-income country
LMIC: low- and middle-income country

## Data Availability

COVID-19 data can be found on the Ministry of Health website [18]. Data on road traffic mortality can be found on the General Statistical Office website [19]. All sources are available to the public and free to access.
